# Mechanical and Fracture Parameters of Ultra-High Performance Fiber Reinforcement Concrete Cured via Steam and Water: Optimization of Binder Content

**DOI:** 10.3390/ma14082016

**Published:** 2021-04-16

**Authors:** Avan Ahmed Mala, Aryan Far H. Sherwani, Khaleel H. Younis, Rabar H. Faraj, Amir Mosavi

**Affiliations:** 1 Department of Civil Engineering, Gaziantep University, Gaziantep 27400, Turkey; avan.mala1@gmail.com; 2 Department of Civil Engineering, Faculty of Engineering, Soran University, Soran 44008, Kurdistan Region, Iraq; aryanfar.abd@soran.edu.iq; 3 Road Construction Department, Erbil Technology College, Erbil Polytechnic University, Erbil 44001, Kurdistan Region, Iraq; khaleel.younis@epu.edu.iq; 4 Civil Engineering Department, Tishk International University, Erbil 44001, Kurdistan Region, Iraq; 5 Civil Engineering Department, University of Halabja, Halabja 46006, Kurdistan Region, Iraq; 6 Faculty of Civil Engineering, Technische Universität Dresden, 01069 Dresden, Germany; 7 Institute of Structural Mechanics, Bauhaus Universität-Weimar, 99423 Weimar, Germany; 8 John von Neumann Faculty of Informatics, Obuda University, 1034 Budapest, Hungary

**Keywords:** mechanical properties, fracture parameters, micro steel fibers, binder content, steam curing, water curing, ultra-high performance fiber reinforced concrete (UHPFRC), silica fume, fracture mechanics, materials design

## Abstract

An investigational study is conducted to examine the effects of different amounts of binders and curing methods on the mechanical behavior and ductility of Ultra-High Performance Fiber Reinforced Concretes (UHPFRCs) that contain 2% of Micro Steel Fiber (MSF). The aim is to find an optimum binder content for the UHPFRC mixes. The same water-to-binder ratio (w/b) of 0.12 was used for both water curing (WC) and steam curing (SC). Based on the curing methods, two series of eight mixes of UHPFRCs containing different binder contents ranging from 850 to 1200 kg/m^3^ with an increment of 50 kg/m^3^ were produced. Mechanical properties such as compressive strength, splitting tensile strength, static elastic module, flexural tensile strength and the ductility behavior were investigated. This study revealed that the mixture of 1150 kg/m^3^ binder content exhibited the highest values of the experimental results such as a compressive strength greater than 190 MPa, a splitting tensile strength greater than 12.5 MPa, and a modulus of elasticity higher than 45 GPa. The results also show that all of the improvements began to slightly decrease at 1200 kg/m^3^ of the binder content. On the other hand, it was concluded that SC resulted in higher mechanical performance and ductility behavior than WC.

## 1. Introduction

Developing in concrete materials can be grouped into three phases. The first phase is conventional concrete that offers normal strength. The second one is high-strength concrete (HSC), which provides higher strength and is a harder material than the former one. The third phase is HSC, which can be distinct with its high compressive strength of almost 70 MPa, flexural tensile strength of nearly 10 MPa, and the modulus of elasticity ranging between 14 and 42 GPa. Nowadays, given the enhancements on the scale of the microscope, reactive powder concrete (RPC) technology is considered a patent in the area of concrete technology known as ultra-high performance concrete [1]. The concept of RPC was first developed by Richard and Cheyrezy [2] Currently, it is considered as Ultra-High Performance Concrete Composite (UHPC). UHPC is considered one of the latest developments in the technology of concrete, and improving many concrete deficiencies [3]. UHPC can be distinct with normal concrete and high performance concrete with its microscale properties of the particle size and the constituent materials [4]. It has been used in many major applications including coupling beams in tall buildings, precast elements, infrastructure repair, and vast facilities such as nuclear waste storage tanks [5,6]. Manufacturing UHPC requires ultra-content of cement and silica fume (SF) as the total binder content for improving the workability, fine grained quartz sand ranged between 150 and 400 µm to reduce the thickness of the paste, low water/binder (w/b) of <0.2, and a special type of superplasticizer (SP) to achieve a workable concrete [7,8,9]. We examined UHPC with a w/b of 0.24, binder amount of 657 kg/m^3^ and a Quarry dust content of 1050 kg/m^3^ using a particle size ranging between 150 μm and 1.18 mm [10]. To attain a workable concrete, superplasticizer was used. The highest rate of compressive strength attained was 122.4 MPa. The main lack of plain UHPC is low in tensile strength versus the high values of compressive strength [11].

When fibers are incorporated, UHPC is called Ultra-High Performance Concrete Reinforced with Fibers (UHPFRC) [12]. Irrespective of its size, type, and shape, the fibers have a key role in enhancing the confrontation of plain UHPC to cracking, ductility, and toughness properties. In UHPFRC, fibers have the potential to promote energy absorption, strain hardening under tension, and to avoid sudden failure [13,14,15,16]. Micro fibers from different resources, including steel, glass, and carbon, are usually employed to produce UHPFRC [17]. For the determination of fracture energy, inverse analysis can be utilized to classify it, but this needs to utilize a particular test program and numerical methods [18]. UHPFRC is usually characterized with ultimate strength, superior fracture parameter, and an enhanced durability if proper manufacturing techniques and curing conditions (water, steam or autoclave curing) are adopted. Steam curing for UHPC is very effective not only because of enhancing the hydration of large amounts of un-hydrated cement inside UHPCs due to large quantities of binder, but it also helps in controlling the moisture movement from and into the concrete [19]. Therefore, researchers preferred steam over water curing for the production of UHPFRCs and superior results were obtained [20,21]. For instance, steam curing of concrete at atmospheric pressure results in a considerable increment in the strength development rate. This method is mainly used for prefabricated concrete parts such as pipes, and prestressed elements; nonetheless, it can also be utilized for closed inlet in situ constructions. In the manufacturing of precast concrete elements, steam curing enables higher production through faster turnover mold and formwork, shorter curing times before shipping, and less product damage during transporting. The pozzolanic reaction, which is thermally activated due to the high curing temperature of steam curing leads to the growth of C-S-H and decrease in calcium hydroxide [22].

The influence of the curing temperature on the properties of cement mortars and concretes was the focus of some studies. It has been largely clarified that curing at a high temperature immediately after casting provides the growth of mechanical characteristics in the initial ages, then adversely affects strength in the final ages. Mouret et al. [23] stated that at 28 days, 10% reduction in compressive strength was detected when the concrete cured at 35 °C related to the concrete cured at 20 °C. Furthermore, with increasing the curing regime from 20 and 50 °C, the strength decreased by up to 28%. This drop down of strength in later age refers to the sudden hydration rate in early ages due to the higher temperature, which delays the consequent hydration and forms a non-uniform distribution of the hydration products [24]. Ho et al. [25] explored the possible benefits of steam curing of concrete with the combination of fly ash, slag and silica fume. It was obtained that mixtures with silica fume (SF) provided a good strength performance and low sorptivity in early ages. Valikhani et al. [26] stated that UHPC at 28 days cured in ambient condition showed a compressive strength of 126 MPa and a tensile strength of 6.5 MPa. Further studies are required to implement different curing methods of UHPC to investigate the development strength at different ages. For this purpose, in the current study, water curing (WC) and steam curing (SC) were applied to produce UHPC based on blended binders such as cement and SF.

The benefits of using silica fume (SF) in concrete mixes include: improving compressive strength, bond strength, and wear resistance; additionally, the resistance against permeability and the corrosion resistance of the embedded are improved [27]. In cementitious matrix, SF is the most widely used amorphous silica. The average particle size of SF is approximately one-tenth that of cement. SF has been utilized in the range of 10–25% by weight of cement since the 1950s due to its Pozzolans and filler properties that make the concrete denser [8]. SF and calcium hydroxide react together to make more C–S–H gel, accordingly improving the final strength [9]. Furthermore, some investigators such as Dunster [10] stated that the contributions of SF and concrete components could minimize cement consumption, which has become sustainable with regards to economic and environmental development.

Various studies have been implemented on the effects of different parameters, including fibers, mineral admixtures and curing condition, on the behavior of UHPFRC. For instance, the effects of various types of fibers on the mechanical performance and ductility behavior cured in water have been studied by researchers [28,29]. For this purpose, a w/b of 0.195 and different types of fiber content ranging between 0.25 and 2% fiber volume fraction were used. It was concluded that the maximum compressive strength was 180 MPa for the micro steel fibers (MSF) and that, for flexural strength, the hooked steel fibers were more effective [30]. Moreover, the effects of nano-silica and micro-silica on the mechanical properties for the 0.2 w/b of UHPC were also investigated by Gesoglu et al. [31]. It was also detected that the binary usage of nano-silica and micro-silica provide superior performance than the individual utilization. Maleka et al. [20] investigates UHPFRC by using blended cement and SF. The content of cement that was chosen in this research was 700, 750 and 800 kg/m^3^ and SF content of 0, 15, and 30% of cement content. The study has recommended the inclusion of the SF of up to 30% of cement content. Also, Yazıcı et al. [32] studied the mechanical properties under different curing regimes containing mineral admixtures. They revealed that the steam and autoclave curing looked like very effective ways to improve the strengths of UHPCs. However, there is very limited research on investigating the optimum content of binder and its effect on the mechanical and fracture parameters of UHPFRC cured via different methods

As a result of reviewing the available literature, the question on how to find the optimum balance between the binder content and aggregate volume to improve the performance of UHPC has become our main concern to find an answer for. Therefore, an experimental program is prepared by taking different volumes of binder ranging between 850 and 1200 kg/m^3^ with an augmentation of 50 kg/m^3^ for investigating strength and fracture parameters of UHPFRC. Moreover, the effect of curing methods (WC and ST) on the UHPFRCs were taken into account. Furthermore, parameters such as flowability of concrete, w/b ratio, SF and MSF content were kept constant unlike the binder content and curing methods (steam curing and water curing) which were varied for optimization purposes of the results of compressive strength, splitting strength, modulus of elasticity, flexural strength, load–displacement curves, fracture energy, and characteristic length.

## 2. Experimental Study

### 2.1. Materials

To implement the experimental study, Portland cement type CEM I 42.5 R and supplementary cementitious materials such as Silica Fume (SF) were utilized as additional cementitious materials (see Table 1). The cementitious materials were provided by local companies from the city of Gaziantep, Turkey. Commercial quartz sand as fine aggregate (specific gravity of 2.65) used with three dissimilar size portions having a particle size in the range of 0–0.4, 0.6–1.2, and 1.2–2.5 mm was utilized as an alternative to the coarse aggregate. Since it is detected that with decreasing the w/b the strength of hardened cement based-materials will increase with decreasing porosity [33,34], the water-to-binder ratio (w/b) for UHPFRC is taken as 0.12 in this study. Type F polycarboxylate-based superplasticizer was used to achieve the workability [35]. To provide fiber reinforcement, micro steel fibers (MSF) were used with 2% volume fraction as shown. Table 2 shows the properties and the aspect ratios of the MSF.

### 2.2. Mixing and Casting

The UHPFRC mix ingredients examined in the current research work are considered with high binder content and 2% of MSFs, eliminate the coarser aggregates, and a very low w/b ratio like other studies [36,37,38,39]. The mixing proportions in this research work comprise two groups, as illustrated in Table 3, depending on curing type (water curing and steam curing) with the same w/b of 0.12. For each mix group, eight mixes of different binder content were casted ranging from 850 to 1200 kg/m^3^ by 50 kg/m^3^ increments as a total cementitious material. The silica fume (SF) was kept constant as 15% of the total weight of the binder for the two groups. In order to adjust the workability of concrete, superplasticizer was utilized in a different rate. The mixes were labeled according to the binder content and curing types. For instance, 950SC specifies the mix holding 950 kg/m^3^ of binder and cured by steam.

The UHPC mixes were produced by a high-speed mixer, the maximum rate of rotating of which reaches up to 470 rpm. The dry ingredients were mixed at 100 rpm for the first 3 min. After the water was added, the batch was mixed at a speed of 100 rpm for an additional 5 min. SP was then fed to the mixed batch and continued at maximum speed for another 5 min. Next, 2% of MSF was added at a lower speed (100 rpm) for 2 min, and the mixed materials resumed for the last 2 min at a maximum speed. Finally, the fresh UHPFRCs were then placed in the molds of different sizes as follows: 3 cubes 50-mm^3^, 3 cubes of 70 mm^3^, 3 cubes of 150 mm^3^, and 3 prisms of 70 mm × 70 mm × 280 mm in dimensions for determining mechanical and fracture parameters of the UHPFRCs. Later, the specimens were compacted by means of vibrating table and then wrapped with a nylon sheet and left in the molds for about 24 h at ambient temperature for the first group. Then, after the specimens were demolded, the first group stored in water that cured at 20 °C until testing date and samples for the second group were subjected to steam curing at 90 °C with a relative humidity of 95% and the cycle took about 48 h and later put in water at 20 °C up to the testing day.

### 2.3. Curing Condition

Two curing methods were implemented in this study for the same series, including water curing (WC) and steam curing (SC). The group of WC-UHPFRC was demolded 24 h after the mixing date and then put in a water tank at 20 °C until the testing age. Conversely, in the condition of SC, UHPFRC samples were put into the camber with the molds and subjected to steam in a fresh state. In the current study, the SC cycle had a total duration of 48 h, accounting for two hours of preheating, three hours of increasing the rate of heating to get the maximum value of 90 °C, and the temperature was constant for 41 h and lastly the chamber was cooled for two hours. The relative humidity in the SC chamber was about 95%. Consequently, the hardened samples were demolded and kept in water at 20 °C up to the testing day.

### 2.4. Testing Methods

**Figure 1:**
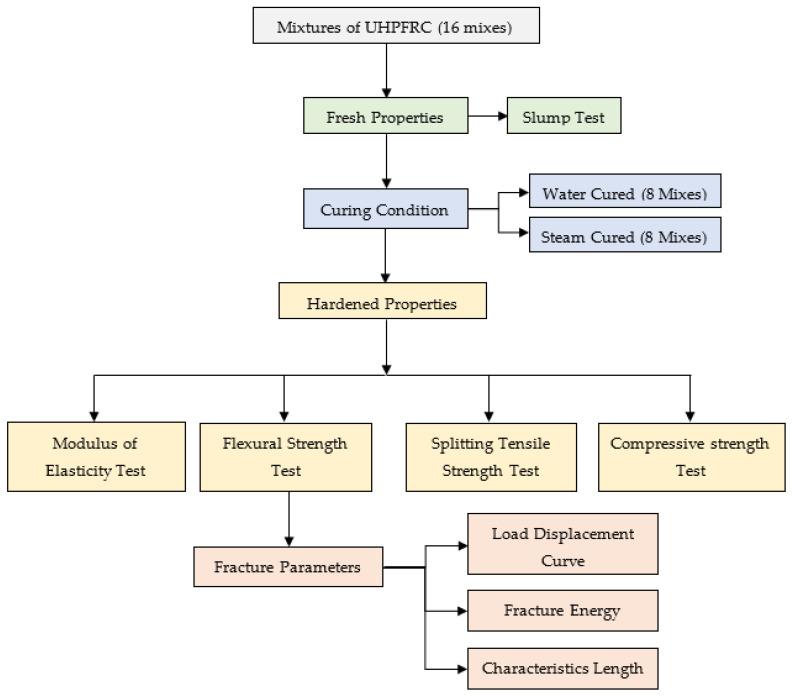
Flow chart diagram for the testing process conducted in this study.

Figure 1 shows the detailed testing process that was conducted in this study. Before the curing process, the mixtures of UHPFRC have been tested for workability, then casted and subjected to curing until the testing date. To implement the compression test, cubic molds of 50 mm were cast and tested at 7 and 28 days following the ASTM C39 [40]. For each reading, an average of three samples was taken. A splitting test was done on the cube of 70 mm at an age of 28 days with respect to ASTM C496 [41]. Meanwhile, a cubic sample of 150 mm was tested at 28 days to measure the modulus of elasticity as specified in ASTM C469 [42]. The specimens were 3 times loaded and unloaded, and the subject load was equal to 40% of the maximum load that could be carried in compression state. The modulus of elasticity for each cube is the average of the second and third set of readings with eliminating the first one.

**Figure 2:**
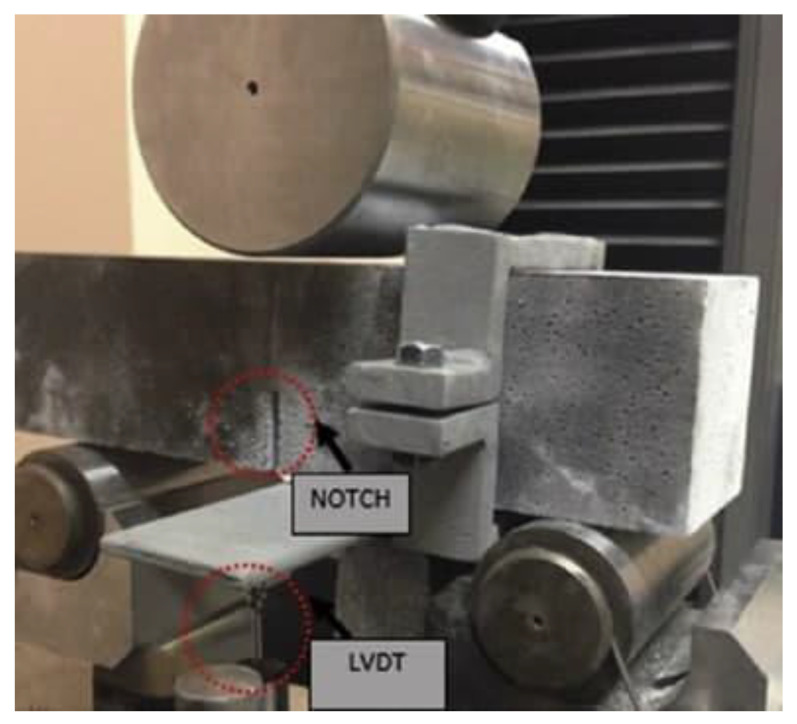
Test details and sample set up.

For evaluating indirect surface energy for the cementitious products, facture energy is used [43]. To carry out the flexural strength test, an average of three prisms of 70 mm × 70 mm × 280 mm was tested with reference of RILEM 50- FMC/198 [44]. A 250 kN capacity closed-loop machine was used for performing the test with a 0.02 mm/minute loading rate. To measure the displacement, a linear variable displacement transducer (LVDT) was fixed at the center of the beam. Figure 2 demonstrates the test details and test set up. Following RILEM [44], measuring the fracture energy (G_F_) of a notched beam by using a three-point loading system can be expressed as shown in Equation (1):

G_F_ = (W_0_ + mgδ_s_(S/U))/B(W − a) (1)

Hence, W_0,_ m, g, and δ_s_ are the area under the load–displacement curve, the beam mass, the gravity acceleration, and the beam deflection, correspondingly, whereas S is span, U is length, B is width, W is depth, and a is the notch depth of the beam. Equation (2) was implemented to calculated the net flexural strength, f_flex_, which the P_max_ is the maximum load considering no notch sensitivity [45,46]. Furthermore, to measure the ductility, the characteristic length (l_ch_) was calculated using Equation (3) which is a function of E, G_F_, and *f*_st_ [47].

f_flex_ = (3P_max_ S)/(2B (W − a)^2^) (2)

l_ch_= (EG_F_)/(*f*_st_^2^) (3)

## 3. Experimental Results and Discussion

### 3.1. Fresh Behavior of UHPFRC

**Figure 3:**
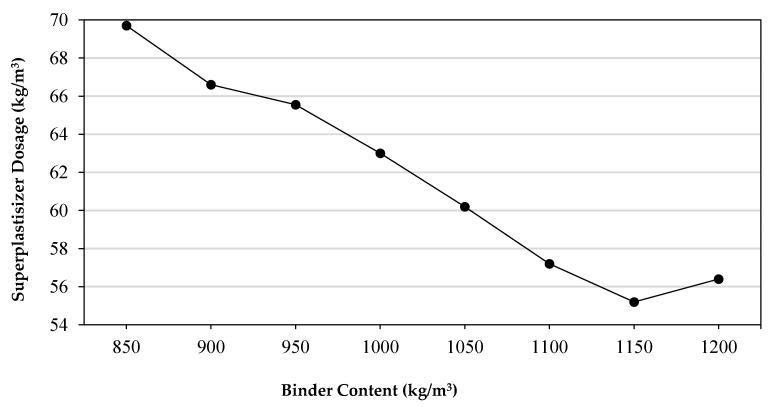
Super plasticizer demand versus binder content.

To achieve high-quality ultra-high performance concrete (UHPC) reinforced with 2% of MSF with the desired workability, the use of superplasticizer (SP) becomes a crucial step due to the low w/b of UHPC. The desired flowability for all mixtures was achieved as kept as 18 ± 1 cm by using a different amount of superplasticizer. The relation between increasing the amount of binder and the corresponding decrease in superplasticizer used is drawn in Figure 3. This inverse relationship may be attributed to the micro-level particles of silica fume (SF), which increased from 127.5 to 172.5 kg/m^3^ responding to an increase in the binder content (850–1150 kg/m^3^). Thus, due to their ultra-fine sizes that areshown in Table 1 compared to cement particles, small spherical silica particles may have helped to increase flowability not decreasing it [48]. In addition, the reason for the necessary increasing of SP with a greater increase in binder after 1150 kg/m^3^ may be attributed to the better dispersion of micro silica particles at 1200 kg/m^3^ of binder used. It was also reported that for the production of UHPC with 2% MSF and 1175 kg/m^3^ binder content at 0.12 w/b the SP demand was nearly 70 kg/m^3^ [28].

### 3.2. Compressive Strength

**Figure 4:**
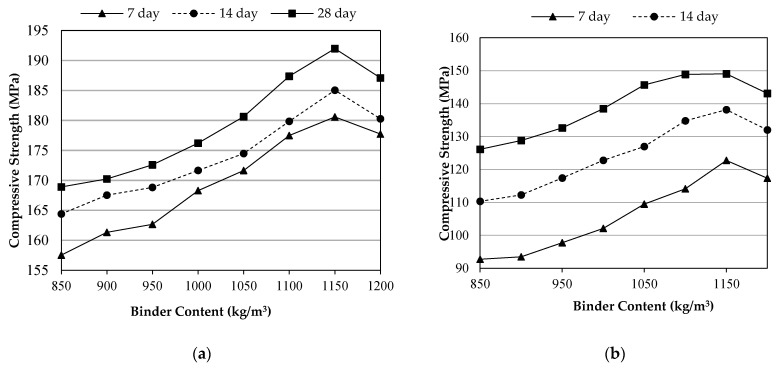
Compressive strength development versus binder content: (**a**) water-curing group; (**b**) steam-curing group.

Figure 4a,b illustrate the compressive strength results of the ultra-high performance fiber reinforced concrete (UHPFRC) versus binder content for the two UHPFRC groups of water curing (WC-UHPFRC) and steam curing (SC-UHPFRC), correspondingly. The compressive strength results of SC concretes were very close to each other, whereas greater differences were noticed for the concretes cured via steam. At 28 days, the compressive strengths were 149 and 192 MPa at 1150 kg/m^3^ binder content as the highest values recorded in this study for the UHPFRCs cured by water and steam, respectively, while the lowest measurements were observed for WC-UHPFRC and SC-UHPFRC at 850 kg/m^3^ of binder content as 129 and 165.5 MPa, respectively. Therefore, steam-cured concretes are superior to water-cured concretes. Likewise, Qadir et al. [28] studied water-cured UHPFRC having 1175 kg/m^3^ binder content with 2% MSF content and 0.12 w/b; a compressive strength of nearly 150 MPa was achieved. Additionally, Yu et al. [49] observed that the use of 2% MSF in the UHPC increased the compressive strength from 99 to 120.8 MPa. Besides, with increasing binder content, lower improvement in the compressive strength was observed for the SC-UHPFRC compared to the WC-UHPFRC as the curing time prolonged. This can be attributed to the high early age strength gaining usually achieved when SC is employed. For example, at the age of 28 days, the 1150WC mixture had a 21.4% increase in compressive strength while the 1150SC had 6.3% strength increments, compared to the 7 days results. Similar results were detected for UHPC with 1175 kg/m^3^ binder and 2% MSF. For 28 days water curing, the compressive strength increment rate was 26%, compared to the 7 days results [28]. The preferable nature of SC than WC is may be because of congestions of high hydration and pozzolanic reaction resulting from the ingredients of the ultra-amount of binder (850–1200 kg/m^3^) and SF (127.5–180 kg/m^3^) that activated dramatically by the high moisture and temperature of curing. Park et al. [50] stated that with increasing the curing temperature, the strength development of the UHPC was enhanced. Moreover, SC samples provide higher strengths compared to WC samples [51]. Regarding the binder content, a significant growth in compressive strength was observed as the binder content increased up to 1150 kg/m^3^, then began to decrease. The faintly lower compressive strength of UHPFRC containing 1200 kg/m^3^ binder may be related to inadequate spreading of the particles of the micro silica in the mix due to their small sizes. The dis-agglomeration of particles is vital to achieve a typical composite material; therefore, the required content of SF in the mixes were exceeded to consume the Ca(OH)_2_ compounds so as to produce more C–S–H gel. Consequently, it no longer influences the strength development of UHPFRC [31].

### 3.3. Splitting Tensile Strength

**Figure 5:**
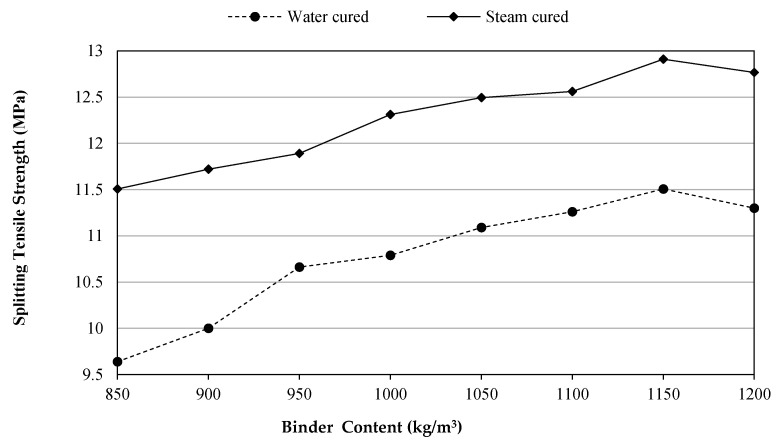
Splitting tensile strength development versus binder content.

Commonly, the utilization of fibers improves the tensile strength of concretes. This might be attributed to the fact that the fibers enable blocking tensile cracks and then followed by limiting crack growth [28]. The variations of splitting tensile strength of UHPFRC with the different amount of binders and two curing conditions are given in Figure 5. It can be noticed that the changes of tensile strength results were dominated due to curing types and different binder content. A remarkable difference in the splitting tensile strength from 9.6 to 11.5 MPa can be observed between the two types (WC to SC) of curing at 850 kg/m^3^ binder content. The splitting tensile strengths for the above two groups (WC and SC) at 1200 kg/m^3^ attained in this study were 11.3 and 12.8 MPa, respectively. Moreover, Gesoglu et al. [30] stated that for the water-cured UHPC reinforced with 2% of MSF at 1200 kg/m^3^ binder content, the splitting tensile strength was about 10.2 MPa. Also, for the micro glass fiber reinforced UHPC at 1175 kg/m^3^ binder content cured via water, the splitting tensile strength was 11.79 MPa [29]. Although, in WC-UHPFRC mixes, the rate of strength development with binder increment is higher than that of SC-UHPFRC, the steam-cured mixes show higher strength values. For example, increasing of binder with increments of 50 kg/m^3^ over 850 kg/m^3^ caused a systematic growth in tensile strength of concretes at 28 days by 3.7%, 10.6%, 12%, 15%, 16.8%, 19.4%, and 17.2% for the water-cured group and 1.9%, 3.3%, 7%, 8.6%, 9.2%, 12.2%, and 11% for the steam-cured group. Besides, adding more binders for the group of SC will not make sense compared to the tensile value gain from WC. This could be due to the hydration process and consuming Ca(OH)_2_ at 850 kg/m^3^ binder content mostly completed and enhanced by high temperature and humidity. Just as in the compression condition, for the UHPFRC specimens it was observed that no vital improvement in splitting tensile strength was attained as the binder content increase beyond the 1150 kg/m^3^, irrespective to the curing type. This might be attributed to the 15% of SF which constitutes 180 kg/m^3^ out of the total binder content of 1200 kg/m^3^, and this quantity of SF could be larger than the quantity needed to consume all cementitious products that resulted from the hydration process, thus leaving some of the SF particles with no chemical reaction [52]. This can also explain the higher content of SP needed to achieve the target slump at 1200 kg/m^3^.

### 3.4. Modulus of Elasticity

**Figure 6:**
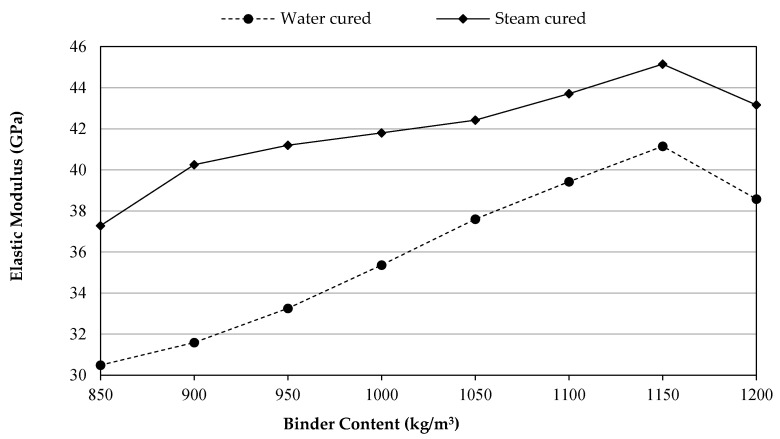
Elastic modulus development versus binder content.

The Young modulus is considered one of the significant material characteristics employed in the design of structural concrete elements since it offers valuable indications about the deformation capacity of concrete in the elastic limit [53,54]. Figure 6 showed the 28 days static elastic modulus of UHPFRC via two curing types and various binder contents. The elastic modulus performance is in line with that observed in the strength results. Based on the increasing rate of binder content, strength development rate in WC-UHPFRC mixes were higher than SC-UHPFRC due to providing a high early age strength at 850 kg/m^3^ by the SC-UHPFRC. Hence, the test measurements revealed that the influence of 1150 kg/m^3^ water cured (1150WC) on the elastic modulus was nearly equivalent to 950 kg/m^3^ of that binder that cured by steam (950SC). This may be attributed to the chemical and physical changes due to temperature and humidity effects on potential for the stress transfer. Additionally, the modulus of elasticity for the water-cured UHPFRC at 1150 kg/m^3^ binder content achieved in this study was 41.15 GPa, whereas the modulus of elasticity for the same MSF content, w/b, and curing condition at 1175 kg/m^3^ binder content achieved by Qadir et al. [28] was nearly 40 MPa.

Furthermore, it was detected that the effect of an increase in the binder content resulted in increasing the concrete’s elastic modulus. The increasing tendency continues until the total binder content reaches up to 1150 kg/m^3^, after which a drop began. At 1150 kg/m^3^ binder content, the UHPFRC had maximum elastic modulus and improved by 35% and 21.1% for the WC and SC groups, correspondingly, when compared to their controls (850 kg/m^3^). It is well recognized that the hydration process has a large impact on the mechanical characteristics of cement-based ingredients, and an appropriate proportion of crystals to non-crystals is preferable to produce higher values [55]. Due to this, an optimum amount of binder can attain an appropriate crystal-to-non-crystal ratio to achieve a greater elastic modulus. Consequently, it is concluded that a huge binder content is disadvantageous to the modulus of elasticity achievement of cement-based materials as observed with the mixtures contained 1200 kg/m^3^ of binder.

### 3.5. Modulus of Rupture (Flexural Strength)

**Figure 7:**
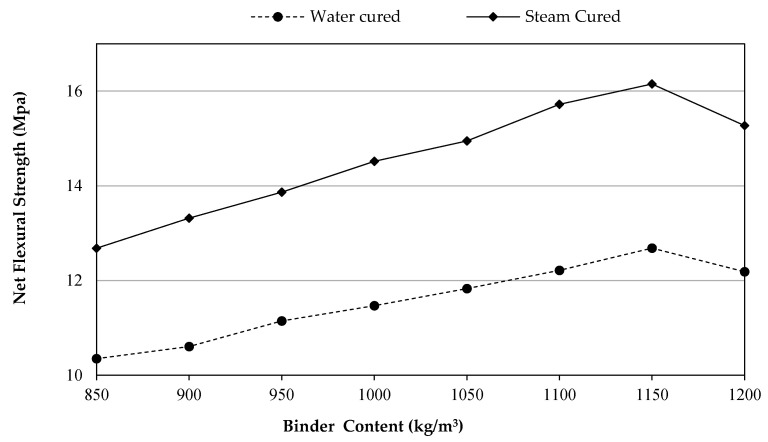
Flexural strength development versus binder content.

The UHPFRC results of the two groups of this experimental research work are in line with the earlier research on improving the flexural strength of UHPCs reinforced with MSF especially at a high rate of binder content [28,29,30]. The effects of binder content on the flexural strength of the UHPCs including 2% of MSF cured via steam and water are illustrated in Figure 7. The preference of SC-UHPFRC over WC0UHPFRC which was noticed from the test results is probably due to the high temperature of steam has motivated the pozzolanic reaction of the calcium hydroxide shaped through the hydration process of cementitious products. In addition, the reaction may take place between unwanted Ca(OH)_2_ and very fine quartz aggregates as well as SF due to hot steam curing conditions [56]. Furthermore, these pozzolanic reactions may lead to a denser C–S–H microstructure that results in an earlier development of strength gaining.

Additionally, it was found that the modulus of rupture (flexural strength) of UHPFRCs continuously increased up to 1150 kg/m^3^ of binder content, irrespective to the curing types. Beyond that, the flexural strength values start to drop down so that utilizing 1200 kg/m^3^ of binder declined the 28days net flexural strength. For example, UHPFRCs with 1150 kg/m^3^ of binder content reached the maximum flexural strength of 12.7 and 16.2 MPa, which reduced to 12.2 and 15.3 MPa, when the total binder reached to 1200 kg/m^3^, for the WC-UHPFRC and SC-UHPFRC groups, respectively. Moreover, for UHPC reinforced with the same content of MSF and 0.195 w/b at 1200 kg/m^3^ binder content, the flexural strength was 8.15 MPa for the 28 days water-cured samples [30]. On the other hand, the reason behind the decline in the flexural strength might be attributed to the amount of binder particles existing in the mixture. They are shown to be greater than the amount necessary to link with the other UHPFRC constituents through the process of hydration. Thus, leading to excess silica leaching out and causing a decrease in strength. Additionally, it may be because of the defects caused in a dispersion of binders that causes weak zones [52].

### 3.6. Load–Displacement Curves

**Figure 8:**
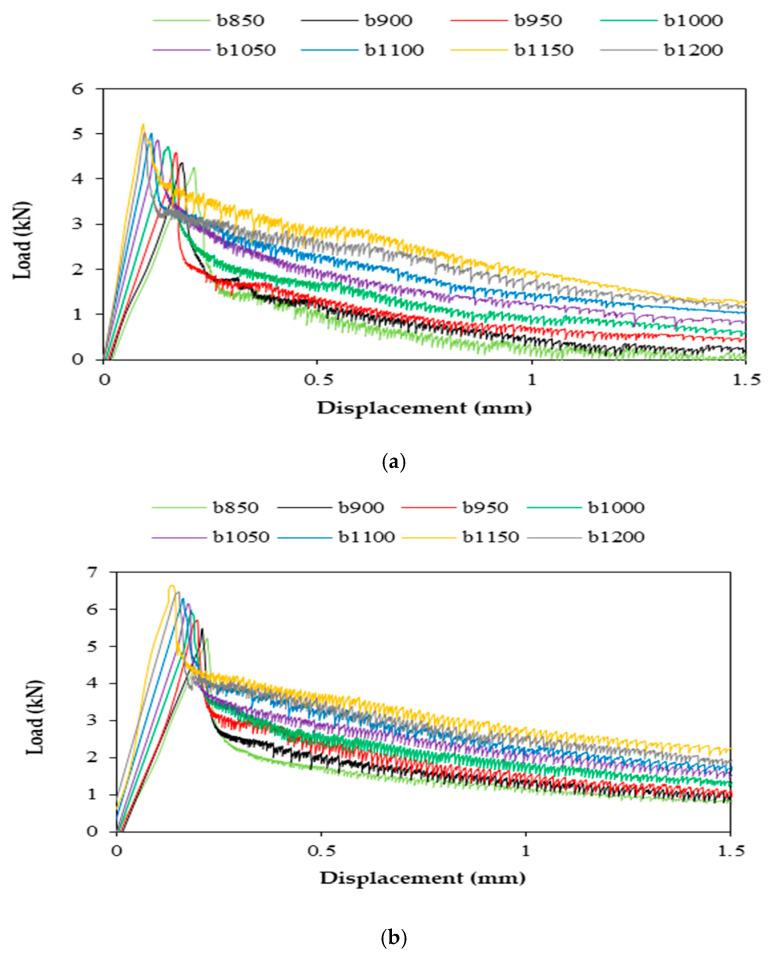
Load versus displacement curves of UHPFRC: (**a**) water-curing group; (**b**) steam-curing group.

The load–deflection curves measured at each loading stage by means of LVDTs for the mixes with different binder amounts of UHPFRC cured via water and steam are given in Figure 8a,b. The LVDTs were set up at the mid-span of the prisms. Regardless of the curing type, the stiffness and toughness are improved via the addition of 2% of MSF in the matrix of UHPC [57]. It can be noted at the initiation of cracking, i.e., at post-peak loads, that the shape of the curves changed into the zigzag form [28]. This may be due to use of MSFs which can significantly tie the micro cracks and enhance the load capacity of the beam specimens when thousands of fibers will stand against the enlargement of cracks. Furthermore, the UHPFRC having 2% MGF with 0.12 w/b at 1150 kg/m^3^ binder content conducted in this study had an area under the curve, a maximum displacement, and a peak load of 4285.1 kN.mm, 4.83364 mm, and 5.2 kN, respectively, for the water-cured group. Similarly, for the same curing condition, w/b, and 1175 kg/m^3^ binder content, the UHPC with 2% micro glass fiber content had an area under the curve, a maximum displacement, and a peak load maximum peak load of 895.5 kN.mm, 1.1 mm, and 6.4 kN, correspondingly [29].

On the other hand, irrespective of the binder content, it can be seen from the abovementioned figures that SC-UHPFRC showed higher performance than WC-UHPFRC, including all important parameters found via load–displacement curves as shown in Table 4. This probably referred to the high-temperature (90 °C) of the steam curing regime provided in a short curing period. Also, it can observed that the peak remarkably depends on the quantity of binder (see Table 4).

Moreover, referring to the binder content, some extent in the pre-peak and post-peak region of the curvature has occurred. The beam load–displacement curves with utilizing a higher binder content dominated a steady decrease in the curve beyond the peak load versus a sharper decrease in the mixtures holding the lower amount of binders. This may be due to the increasing of energy required for debonding UHPFRC components that contained a high volume of binders. Furthermore, the pre-peak and the early post-peak areas in the curves essentially influenced the microcracks and the crack development. Whereas the decrease in the slope at the end of the softened branches could be due to the degree of interlocks between aggregates and fiber content [58], where the binder amounts have a relatively significant role in both phenomena.

### 3.7. Fracture Energy

**Figure 9:**
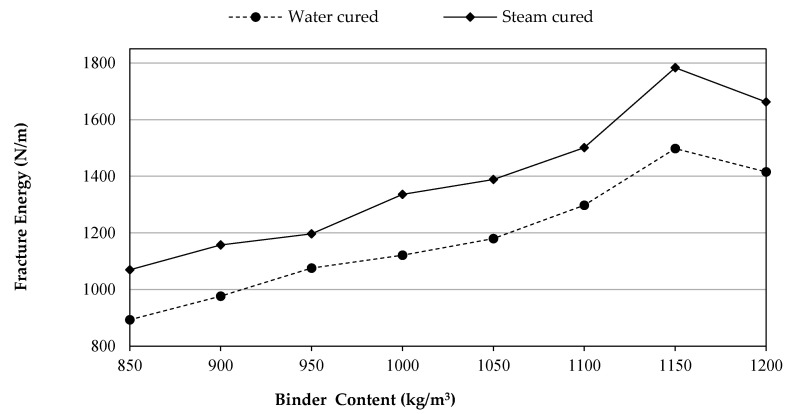
Fracture energy development versus binder content.

The fracture energy (G_F_) is distinct as the material’s capacity to absorb the energy in the post-crack region, and it can be characterized to absorb the energy by the material for the period of failure. Conversely, the ductility behavior of concrete is specified by the fracture parameters, the high value of G_F_ refers to the high the ductile behavior of the concrete [30,31]. In this experimental work, the G_F_ values of UHPFRC versus different binder content for all the mixes of WC and SC are presented in Figure 9. With regard to the curing types, the effect of SC on GF is superior than water curing because steam curing boosts the pozzolanic reaction, improves the microstructure of the compounds, and consumes Ca(OH)_2_ rapidly; thus, it enhances the fracture energy greatly compared to the results of the WC groups.

Furthermore, it is estimated, similar to the aforementioned strength test results, that the UHPFRC will improve significantly with increasing binder content up to 1150 kg/m^3^ in which the maximum values were recorded, beyond that limit the GF values were declined, irrespective of the curing types. Additionally, the development rate of fracture parameters for the UHPC is greater than other mechanical properties including compressive strength, tensile strength, and modulus of elasticity. This is due to the fact that fracture properties mostly rely on bond strength and arresting cracks, which can be improved by utilizing the high volume of MSF and ultra binder content, whereas the other properties depend on improving the interfacial transition zone (ITZ) that fibers do not contribute directly to improve [28]. The GF values improved by 9.3, 20.5, 25.6, 32.1, 45.3, 67.7, and 58.5 % as well as 8.2, 11.9, 25, 29.9, 40.4, 66.8, and 55.5 % for the WC and SC groups of UHPFRC, correspondingly, compared to their reference mixtures (850 kg/m^3^) at 28 days, when 900, 1000, 1050, 1100, 1150 and 1200 kg/m^3^ of binder were combined. The main reason behind this enhancement in GF may be attributed to the filling properties of SF, when their amount increased from 127.5 to 180 kg/m^3^ responding to change in the binder content from 850 to 1200 kg/m^3^. Consequently, the ITZ and cement paste may have a strength development, then cracks were desired to pass through the aggregate particles rather than the ITZ and cement matrix [31]. Besides, the reason behind the adverse performance of UHPFRCs after 1150 kg/m^3^ (at 1200 kg/m^3^) may be related to the fact that there is a much greater necessity amount of silica inside concrete, and extra silica will leach out, resulting in forming weak zones through the matrix. This could be the reason for the decline in the values of Gf at 1200 kg/m^3^ as can be seen in Figure 9.

### 3.8. Characteristic Length, $l_{\mathrm{ch}}$

Characteristic length (l_ch_) is a decent property to evaluate the brittleness of concrete materials. Concrete is expected to have less brittleness whenever its l_ch_ is high. As presented in Equation (3), the characteristic length of concrete is directly proportional with fracture energy and modulus of elasticity, whereas it has an inverse relation with tensile strength.

**Figure 10:**
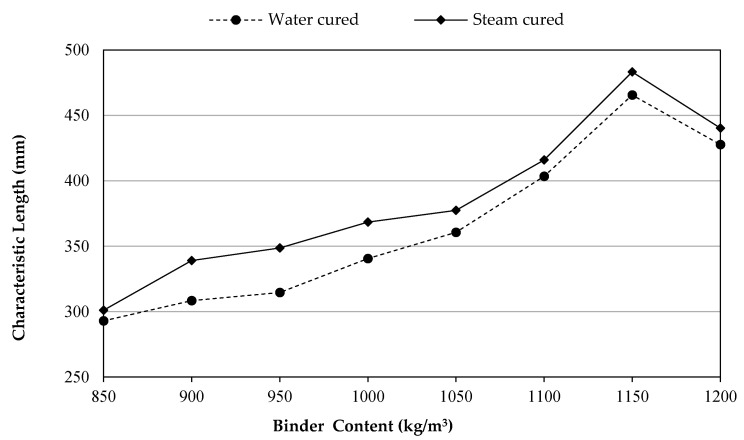
Characteristic length development versus binder content.

The characteristic length values of the UHPCs including 2% of micro steel fibers are illustrated in Figure 10 with respect to the different binder contents that were exposed to two curing groups of water and steam. For instance, the l_ch_ for the water-cured samples at 1150 kg/m^3^ attained in this study was 483 mm. Besides, the l_ch_ for the micro steel fiber reinforced UHPC that cured via water at 1175 kg/m^3^ binder content was about 580 mm [28]. Based on the curing types, it was observed that the differences were minimal, as is noticeable from the ductility results shown in the above figure; however, steam curing achieved higher values than water curing. The occurrence of such behavior could be due to the fact that the hydration process probably is more activated and continued for a period of time in the steam curing condition. In addition, the pozzolanic reaction resulting from the ingredients of SF in the UHPFRC mixtures will be enhanced energetically by the high moisture and temperature of curing, and it may directly affect the brittleness of concrete.

Alternatively, the effects of increasing binder from 850 up to 1150 kg/m^3^ seemed to be significant on increasing the ductility ‘decreasing brittleness’ of UHPFRCs as the l_ch_ values range between 293 and 465.5 mm and 301.1 and 483.2 mm for the water and steam curing groups, respectively; then, after adding more binder (1200 kg/m^3^), the tendency for brittleness increased. This behavior of enhancement of ductility may be referred to the binary effects of the high volume content of SF and cement accompanying the very low w/b of 0.12, thus leading to converting material being more ductile. The reason for concrete’s tendency for brittleness at 1200 kg/m^3^ could be because of the fact that any extra amount of SF more than 172.5 kg/m^3^, which contributes 15% of the weight of the total binder of 1150 kg/m^3^, may work as a lubricant area to slippage the other concrete constituents over each other. There is a gap of research on the characteristic length of UHPC, but many reports have tackled it for conventional concretes. For instance, for compressive strengths that range between 40 and 80 MPa, the l_ch_ was measured by Zhang et al. [59] that ranged from 412 to 235 mm, and it was also ranged from 200 to 500 mm by Petersson [60]. Additionally, for the high strength self-compacting concretes including plastic, Faraj et al. [61] stated that l_ch_ is in the range of 85 to 178 mm.

## 4. Conclusions

In this experimental research work, the impact of different binder content (comprising 85% cement + 15% SF) and curing types on the performance of UHPC reinforced with 2% MSF at 0.12 w/b has been observed. The findings attained in this research can be summarized as follows:

1. An inverse relation occurred with an increasing amount of binder via super plasticizer. This may be attributed to the increase in SF responding to the increase in the binder content. Moreover, the reason for the necessary increasing of SP with a greater increase in binder after 1150 kg/m^3^ may be attributed to the dispersion of SF at 1200 kg/m^3^ of binder content.
2. In the present study, the performance of SC-UHPFRC was much better than WC-UHPFRC. Therefore, better results were achieved for all the measured properties conducted in this study for the mixes cured by steam. This might be because of the potential of high hydration and the pozzolanical reaction of the ultra-amount of binder (850–1200 kg/m^3^) that was activated by the high moisture and temperature of curing.
3. SC-UHPFRC mixes had considerable high early strength compared to WC-UHPFRC. However, based on the binder content, it was observed that WC-UHPFRC had a significantly higher rate of strength development.
4. It was observed that there was a systematic growth in the mechanical properties and ductility behavior of UHPFRC with increasing the binder contents up to 1150 kg/m^3^ and then the results were dropped. This behavior of UHPFRC containing 1200 kg/m^3^ binder could be because of the inadequate spreading particles of SF or it could be due to the fact that the increase in SF content has no more role in forming C–S–H gel as all CH compounds have been consumed.
5. The highest compressive strengths noticed at 1150 kg/m^3^ of binder content were 149 and 192 MPa, while the lowest were 129 and 165.5 MPa at 850 kg/m^3^ of the binder amount for the WC-UHPFRCs and SC-UHPFRCs conditions, respectively.
6. Increasing the binder content with an increment of 50 kg/m^3^ over 850 kg/m^3^ caused a systematic growth in the splitting tensile strength of concrete by 3.7%, 10.6%, 12%, 15%, 16.8%, 19.4%, and 17.2% for the WC-UHPFRC group and 1.9%, 3.3%, 7%, 8.6%, 9.2%, 12.2%, and 11% for the SC-UHPFRC group.
7. The test results of UHPFRC revealed that the influence of 1150 kg/m^3^ of binder cured by water (1150WC) on the elastic modulus was almost equivalent to 950 kg/m^3^ of binder content cured by steam (950SC).
8. The flexural strength of UHPFRC with 1150 kg/m^3^ binder had the highest values of 12.7 and 16.2 MPa then reduced to 12.2 and 15.3 MPa at 1200 kg/m^3^ binder content, for the WC-UHPFRC and SC-UHPFRC, respectively.
9. The toughness of the UHPFRC was improved with using more binder content as the area under the curve and the peak load were increased. SC-UHPFRC provided higher values than WC-UHPFRC. Moreover, at the post-peak region in the load–displacement curves, the curves have a zigzag form. Such form might have occurred because of the mechanism of micro cracks due to the crack-bridging process over the micro steel fibers.
10. More ductile UHPFRC can be achieved with increasing the binder content, irrespective of the curing types. The optimum values of UHPFRC were obtained at 1150 kg/m^3^ binder content. For instance, the GF improved by 67.7% and 66.8% for the water- and steam-cured groups, respectively, compared to their reference mixtures (850 kg/m^3^). Beyond that binder content, GF values decreased.
11. The range of the characteristic length of UHPFRC was between 293 and 465.5 mm and 301.1 and 483.2 mm for the binder content ranging from 850 to 1150 kg/m^3^ for the concretes cured via water and steam, respectively. Adding more binders (1200 kg/m^3^) increases the tendency of concrete to be brittle.

## Figures and Tables

**Table 1:** Physical and chemical characteristics of Portland cement and silica fume.

Constituent (%)	PC	SF
CaO	57.87	0.45
SiO_2_	17.99	90.36
Al_2_O_3_	3.88	0.71
Fe_2_O_3_	3.36	1.31
MgO	1.49	-
SO_3_	2.47	0.41
K_2_O	-	1.52
Na_2_O	-	0.45
Cl	0.005	-
Loss of Ignition	3.37	3.11
Insoluble Residue	0.34	-
Free CaO	2.18	-
Specific surface (m^2^/kg)	394 ^a^	21,080 ^b^
Specific gravity	3.15	2.2

^a^ Blaine specific surface area. ^b^ BET specific surface area.

**Table 2:** Aspect ratio, physical and mechanical properties of MSF.

Type	Length (L) (mm)	Diameter (d) (mm)	Aspect Ratio (L/d)	Density (g/cm ^3^)	Tensile Strength (N/mm^2^)
MSF	6	0.16	37.5	7.17	2250

**Table 3:** Mixing proportions of UHPFRC in kg/m^3^.

Mix Group	Code	Binder Content	w/b	Cement	SF	Water	MSF (%)	Aggregate
Water-Cured Group (8 mixes)	850WC	850	0.12	722.5	127.5	102	2	1365.8
	900WC	900	0.12	765	135	108	2	1312.6
	950WC	950	0.12	807.5	142.5	114	2	1254.4
	1000WC	1000	0.12	850	150	120	2	1199.9
	1050WC	1050	0.12	892.5	157.5	126	2	1145.9
	1100WC	1100	0.12	935	165	132	2	1092.5
	1150WC	1150	0.12	977.5	172.5	138	2	1036.6
	1200WC	1200	0.12	1020	180	144	2	972.9
Steam-Cured Group (8 mixes)	850SC	850	0.12	722.5	127.5	102	2	1365.8
	900SC	900	0.12	765	135	108	2	1312.6
	950SC	950	0.12	807.5	142.5	114	2	1254.4
	1000SC	1000	0.12	850	150	120	2	1199.9
	1050SC	1050	0.12	892.5	157.5	126	2	1145.9
	1100SC	1100	0.12	935	165	132	2	1092.5
	1150SC	1150	0.12	977.5	172.5	138	2	1036.6
	1200SC	1200	0.12	1020	180	144	2	972.9

**Table 4:** Load–displacement parameters of UHPFRC.

CODE	Area under the Curve	Maximum Disp.	P_max_
	W_o_	δ_s_	kN
850WC	2487.0	5.64731	4.3
900WC	2744.0	5.14114	4.4
950WC	3039.0	5.00372	4.6
1000WC	3150.0	6.08267	4.7
1050WC	3340.0	5.32244	4.9
1100WC	3697.5	4.7947	5.0
1150WC	4285.1	4.83364	5.2
1200WC	4038.6	4.98909	5.0
850SC	3009.0	5.47963	5.2
900SC	3274.0	5.17962	5.5
950SC	3387.9	5.27415	5.7
1000SC	3799.3	5.23746	6.0
1050SC	3951.1	5.34784	6.2
1100SC	4275.7	5.54869	6.5
1150SC	5116.1	5.09704	6.6
1200SC	4766.4	4.90861	6.3

## Data Availability

Data can be obtained from corresponding authors upon reasonable request.
